# Induction of remission with tacrolimus in a patient with severe acute, cortisone refractory ulcerative colitis and severe Covid-19 pneumonia: a case report

**DOI:** 10.1186/s12876-022-02094-3

**Published:** 2022-01-15

**Authors:** Lisanne Rieker, Johannes Hofer, Golo Petzold, Volker Ellenrieder, Ahmad Amanzada

**Affiliations:** Clinic for Gastroenterology, Gastrointestinal Oncology and Endocrinology, University Medical Center Goettingen, Robert-Koch-Str. 40, 37075 Goettingen, Germany

**Keywords:** Ulcerative colitis, Inflammatory bowel disease, Case report, Covid-19, SARS-CoV-2

## Abstract

**Background:**

Therapy regimens used in patients with inflammatory Bowel Disease (IBD) have been associated with enhanced risk of viral infections or viral reactivation. Moreover, it is uncertain whether IBD patients have increased risk of severe acute respiratory syndrome coronavirus 2 (SARS-CoV-2) infection or infected patients may have an increased risk for severe coronavirus disease 2019 (Covid-19). Managing severe acute flare in ulcerative colitis during the Covid-19 pandemic is a challenge for clinicians and their patients. The results of the published studies mainly report on the role of the prior medication, but not how to treat severe acute flare of IBD patients with severe Covid-19 pneumonia.

**Case presentation:**

We report the case of a 68-year-old patient with a long history of ulcerative colitis. He was initially admitted to an external hospital because of severe acute flare. The initiation of a high-dose oral cortisone therapy did not improve the clinical symptoms. During the inpatient treatment, he was tested positive for SARS-CoV-2. At admission to our hospital the patient showed severe flare of his ulcerative colitis and increased Covid-19 symptoms. A cortisone-refractory course was noticed. After detailed multidisciplinary risk–benefit assessment, we initiated an intravenous tacrolimus therapy and dose of prednisolone was tapered gradually. After clinical response, the therapy was adjusted to infliximab. Additionally, the Covid-19 pneumonia was kept under control despite immunosuppression and the patient could be discharged in clinical remission.

**Conclusions:**

This case suggest the use of tacrolimus as a bridging therapeutic option for severe acute, cortisone refractory ulcerative colitis in Covid-19 patients. Nevertheless, the best treatment strategy for IBD patients presenting a flare during the outbreak has yet to be defined. Further data for IBD patients under calcineurin inhibitor therapy are urgently needed.

## Background

Patients with severe acute ulcerative colitis suffer of diarrhea with bloody stools (≥ 6 per day) and markers of systemic toxicity as defined by the Truelove and Witts criteria [1]. These patients require admission to hospital for their acute severe flare [2, 3]. Before the coronavirus disease of 2019 (Covid-19) pandemic severe acute ulcerative colitis was associated with a mortality rate of 1.0–2.9% [2, 4].

During the first wave of the Covid-19 pandemic results of small cohort studies suggested that disease activity in inflammatory bowel disease (IBD) patients might be a predictor for adverse Covid-19 outcomes [5, 6]. Regarding nosocomial spread of severe acute respiratory syndrome coronavirus 2 (SARS-CoV-2) infection, especially in those thought to be vulnerable to severe Covid-19 outcomes, physicians might have used a higher clinical threshold to determine which patients required emergency hospital admission. Moreover, early endoscopic assessment may have been affected by uncertainty and delays regarding preendoscopic viral screening, staffing shortages, endoscopic capacity and availability of personal protective equipment.

Inconsistent study results regarding the effect of high-dose steroids in SARS-CoV-2 infection or Covid-19 required conventional steroid treatment dosing strategies [7, 8]. Data are emerging for discussion and decision concerning the risk to benefit ratio of drugs used as rescue therapy, i.e. infliximab, ciclosporin A or tacrolimus [8, 9].

The impact of possible changes to conventional treatment options for acute severe ulcerative colitis outcome are currently unknown. Several of the current recommendations relating to IBD care during the Covid-19 pandemic, including acute severe ulcerative colitis, are only based on expert consensus supported by rare published data [10, 11]. Normally, the treatment of choice in patients with severe acute flare of ulcerative colitis is administration of an intravenous corticosteroid. In the case of corticosteroid refractory course a switch in the treatment regimen is needed, i.e. the application of infliximab or a calcineurin inhibitor and an approach for colectomy.

The growing number of Covid-19 infections is leading to an overlap with other diseases and can cause difficulties in finding therapies. Therefore, this case report describes the diagnosis and successful treatment with tacrolimus followed by infliximab of a patient with cortisone refractory severe acute ulcerative colitis and simultaneous symptomatic Covid-19 infection.

## Case presentation

A 68-year-old male patient was initially admitted to an external hospital because of severe acute activity of ulcerative colitis with high-frequency bloody diarrhea on February 01th 2021. In 1984, a left-sided ulcerative colitis was diagnosed. Subsequently, in the following years a psoriatic arthritis and sacroiliitis were diagnosed. He initially treated with systemic and locally 5-aminosalicylic acids. In the event of intermittent relapses, steroid treatment was established, which resulted in remission. A basic therapy with azathioprine was initiated with a gradual reduction of the steroid dose but had to be terminated due to hepatotoxicity. There were further drug changes to adalimumab, methotrexate and leflunomide. At the time of being hospitalized, he was only under medication with prednisolone 5 mg daily.

**Fig. 1:**
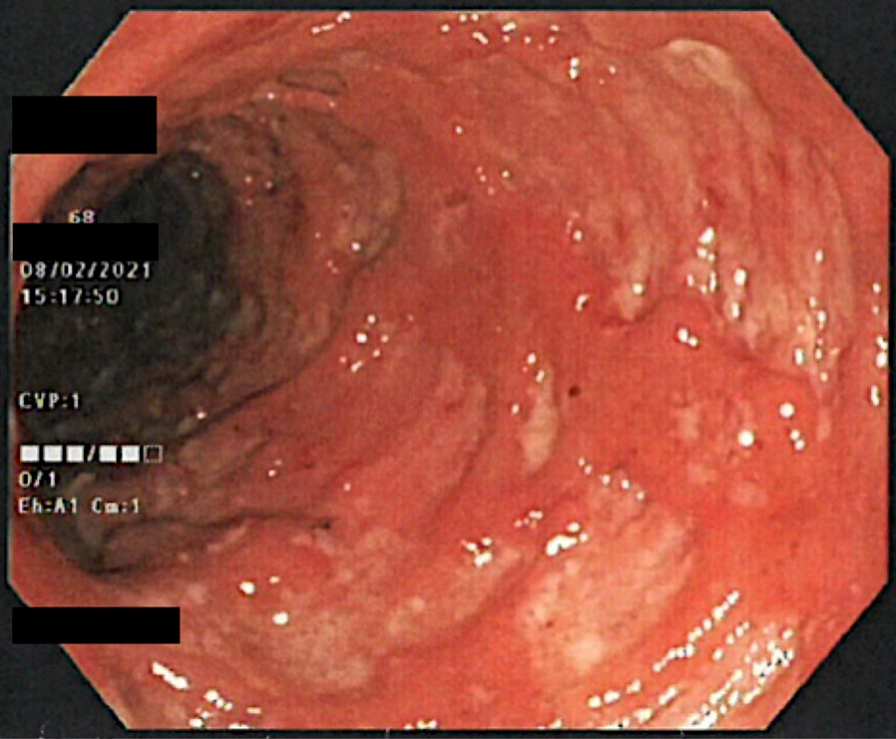
The picture presents the colonoscopy findings with the severe inflammatory activity localized in the sigmoid colon

A colonoscopy was performed and a continuous pronounced inflammation from rectum up to descending colon was detected (Fig. 1). Histology showed a florid ulcerative inflammation, cytomegalovirus infection could be ruled out. Furthermore, fecal tests excluded clostridioides difficile as well as other enteric bacterial or viral pathogens. Oral prednisolone therapy 60 mg daily was initiated.

16 days after hospitalization, the patient was tested positive for SARS-CoV-2 with the variant Wuhan after initial negative polymerase chain reaction. Except for mild cold symptoms without fever, he did not suffer from any other symptoms typical for Covid-19 at that time. Due to the simultaneous infections and the resulting high risk of poor disease progression, the patient was transferred to our unit at University Medical Center Goettingen on February 22nd. At admission to our hospital the patient was in poor general condition with prerenal acute-on-chronic kidney failure.

**Fig. 2:**
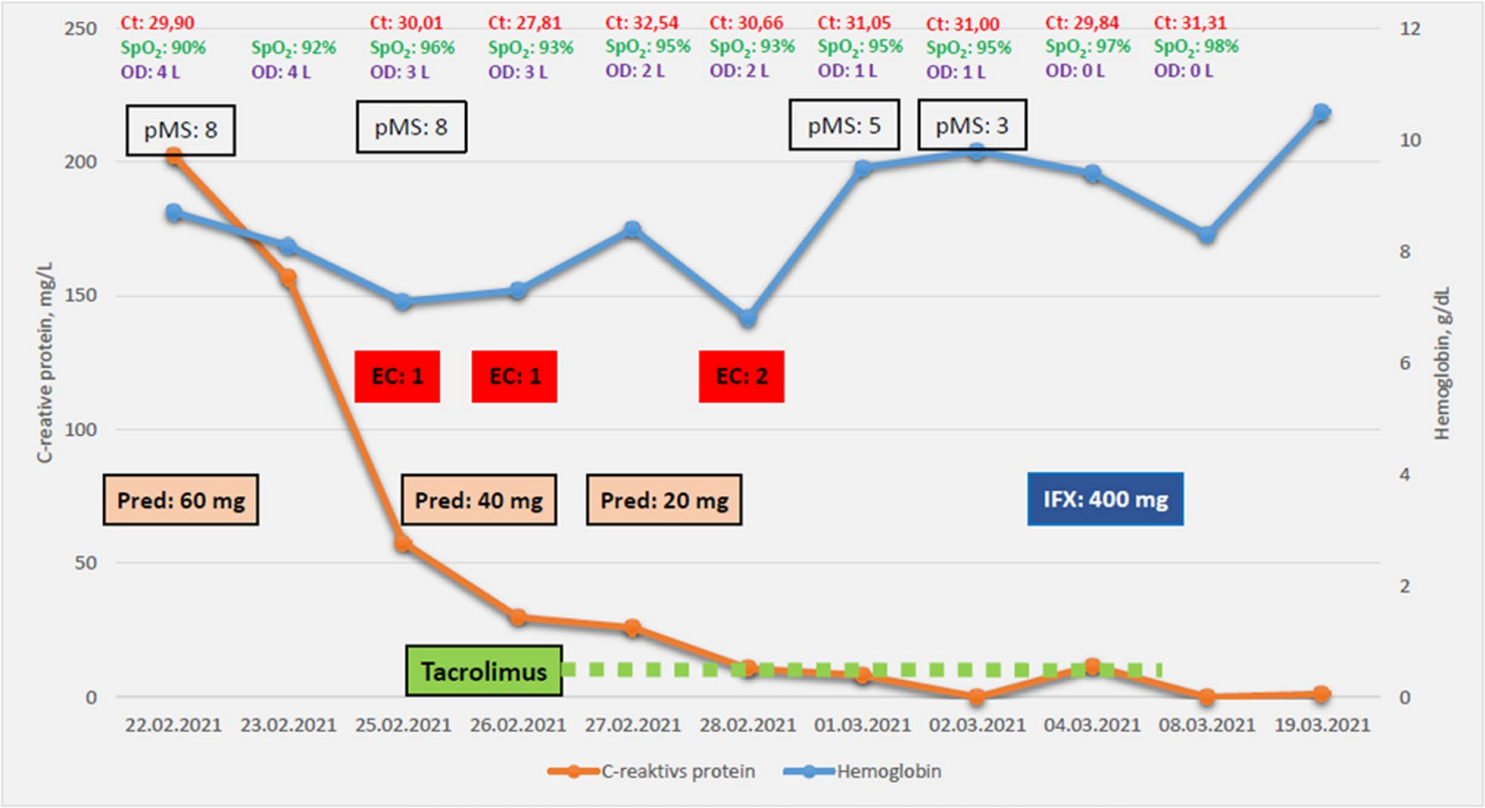
The graphic shows the course of C-reactive protein and hemoglobin. On the top of the graphic are presented the Crossing threshold (Ct) value of SARS-CoV-2 (red), oxygen saturation (SpO_2_, green) and oxygen demand in Liter per minute (purple). Partial Mayo-score (pMS) demonstrates the clinical activity signs. EC (highlighted in red) shows the transfusion of red blood cell concentrates, tapering of prednisolone dose (Pred:…; highlighted in light orange) was made as stated. Begin and end of tacrolimus therapy was initiated as stated (highlighted in green). Administration of infliximab (IFX) is highlighted in dark blue

**Fig. 3:**
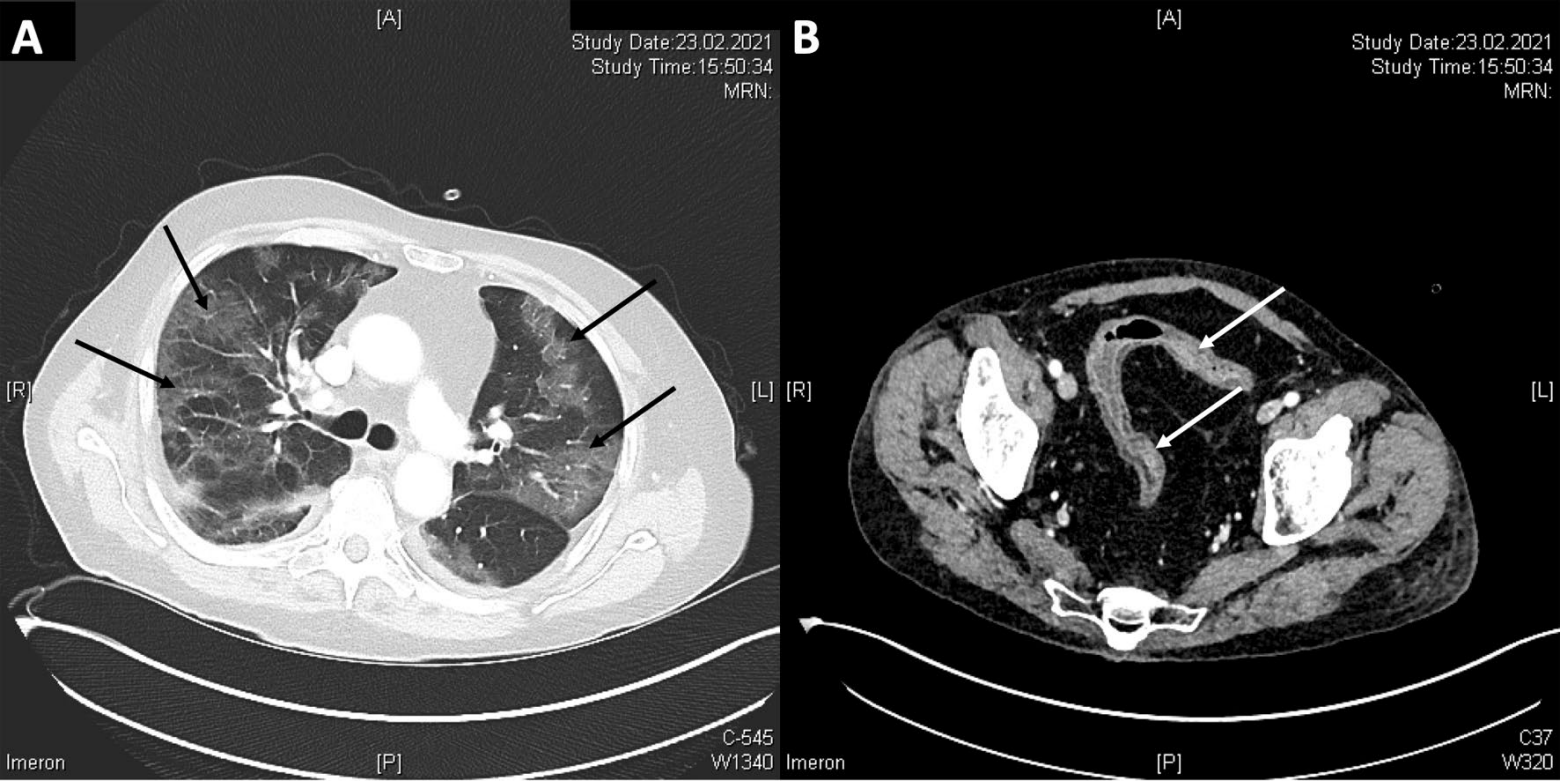
Computer tomography scan showed (black arrows) diffuse ground glass opacities in both lungs (**A**) and a pronounced bowel wall thickening (white arrows) of sigmoid colon (**B**)

Initial laboratory findings showed an anemia (hemoglobin 8,1 g/dl; normal value: 13.5–17.5) and elevation of C-reactive protein (202.4 mg/l; normal value: < 5 mg/l) (Fig. 2). The values of procalcitonin (1.02 µg/l; normal value: < 0.07) were elevated with normal number of leucocytes. Albumin levels were low with 1.7 g/dl (normal value: 3.4–5.0). Due to the elevated levels of D-dimers (1.14 mg/l; normal value: < 0.50), we performed a thoracic computer tomography scan with angiography. Pulmonary embolism could be ruled out, but inflammatory infiltrates in accordance with a Covid-19 pneumonia and mild pleural effusions (Fig. 3A) could be detected. Furthermore, abdominal computer tomography scan showed a wall thickening of the sigma, but ruled out an abscess (Fig. 3B).

Regarding the Covid-19 infection, the patient developed acute hypoxemic respiratory failure during the night after takeover to our hospital. The initial Crossing-threshold-value of SARS-CoV-2 was 29.90. The oxygen saturation was 90% and a supply of 4 Liter oxygen per minute using a nasal cannula was necessary (Fig. 2). Thereafter, the oxygen therapy had to be continued for a total of 8 days. Auscultatory findings were bilateral basal crackles over the lungs. Cough or fever did not occur, the patient's sense of smell and taste were still retained. Due to the National Institutes of Health his Covid-19 pneumonia was classified as severe (https://www.covid19treatmentguidelines.nih.gov/overview/clinical-spectrum/).

Initially, the therapy was switched to intravenous prednisolone 60 mg daily over 4 days. There was a drop in CRP and procalcitonin values, but also of hemoglobin. Unfortunately, up to 10 bloody bowel movements per day persisted under therapy, why the patient required four transfusions of red blood cell concentrates.

After detailed multidisciplinary risk–benefit assessment, we changed the immunosuppressive treatment by initiating intravenous therapy of tacrolimus daily in a dose of 0.01 mg/kg bodyweight over the time of 12 h on February 26th. Simultaneously, the dose of prednisolone was tapered gradually. According to symptomatic response, we continued tacrolimus therapy under drug monitoring (trough level: 4.0–15.0 µg/l). As the gastrointestinal symptoms were controlled, therapy was adjusted to infliximab with first administration of 400 mg on March 5th.

Laboratory parameters regarding inflammation declined significantly over the course of the patient’s hospitalization (Fig. 2). Additionally, the Crossing-threshold-value—reflecting the SARS-CoV-2 virus load in nasopharyngeal swabs—remained continuously detectable but stable indicating that Covid-19 infection was kept under control despite immunosuppression. In addition, SARS-CoV-2 antibodies were detected serologically. On March 9th the patient could be released in clinical remission. The 2nd and 3rd infliximab infusions were administered on an outpatient setting and the patient was still in clinical remission.

## Discussion and conclusion

Physicians are faced with a dilemma in finding the best medical treatment for patients with simultaneous occurrence of severe activity of an IBD and Covid-19 pneumonia. This case report demonstrates that immunosuppressive therapy can be effective and safe despite active Covid-19 pneumonia. In this corticosteroid refractory severe acute flare of ulcerative colitis tacrolimus was initiated because of the high effectiveness, short half-life, and good controllability of this calcineurin inhibitor. Moreover, the advantage of calcineurin inhibitors over tumor-necrosis-factor-α (TNF-α) inhibitors is their lower perioperative complication rate. Research on other subtypes of human coronaviruses prior to the pandemic outbreak in 2019 suggests the need for immunophilin-dependent signaling pathways for coronavirus growth. Immunomodulator tacrolimus was able to suppress virus replication of coronaviruses in cell culture [12]. In liver-transplanted patients with SARS-CoV-2 infection, the latest data indicate a lower mortality under tacrolimus based immunosuppression compared to other immunosuppressants [13].

Advanced age is the main risk factor for a severe course of Covid-19. Moreover, obesity, male gender and various comorbidities have been identified as further risk factors regarding a worse outcome. This also includes the presence of an immunodeficiency or immunosuppressive therapy such as steroids in doses of more than 20 mg/day [14–16]. Last mentioned aspect and the resulting clinical challenge of choosing the best possible treatment with limited data was described in the present case. It shows the successful treatment of a patient with a severe, steroid-refractory episode of his known ulcerative colitis and simultaneous Covid-19 pneumonia. Initially, the values of CRP and procalcitonin were elevated which could possibly indicate bacterial involvement. Moreover, both diseases have probably contributed to the increased inflammatory parameters. After switching to intravenous cortisone therapy, the inflammatory markers improved significantly and symptoms of Covid-19 did not aggravate. The escalation of drug regimen tacrolimus with consecutive switch to infliximab led to rapid improvement of colitis specific symptoms as well as respiratory insufficiency. The oxygen therapy could be reduced gradually.

Furthermore, the important role of the cytokine TNF-α in the pathogenesis of IBD on the one hand and in the inflammatory phase of Covid-19 pneumonia on the other hand has to be considered [17]. Recently published studies suggested that monotherapy with TNF-α antagonists could have a protective effect against severe Covid-19 infection [8, 18]. In analogy to an already published case report discussing treatment with anti-TNF-α in a patient suffering from active ulcerative colitis and simultaneous Covid-19 [19], the activity of both diseases could be suppressed sustainably in the here presented case of a 68-year-old male patient. Two weeks after infliximab was first administered, the SARS-CoV-2 virus could no longer be detected in the nasopharyngeal swab.

To further examine the optimal treatment, more data from more cases still need to be collected. It is yet too early to derive reliable evidence from it. Over time and with a larger patient collective, it will become clearer whether immunosuppressive therapy in IBD patients is more of a “blessing” or a “curse” concerning the outcome of SARS-CoV-2 infection. However, there are reasons to assume that immunomodulatory treatment could be justifiable for mild to moderate and even severe Covid-19 disease.

In the case discussed above, it can be concluded that the patient had a good benefit regarding his ulcerative colitis and no aggravation of his Covid-19 infection. Therefore, short-term tacrolimus therapy under controlled conditions could represent an alternative therapeutic regimen for patients with severe acute flare of cortisone refractory ulcerative colitis and simultaneous Covid-19 pneumonia.

## Data Availability

All data generated or analyzed during this study are included in this published article.

## Abbreviations

IBD: Inflammatory bowel disease
SARS-CoV-2: Severe acute respiratory syndrome coronavirus 2
Covid-19: Coronavirus disease 2019
TNF-α: Tumor-necrosis-factor-α
